# A Chapter of Accidents

**Published:** 1856-07

**Authors:** R. Thompson

**Affiliations:** Nashville, Tenn.


*A Chapter of Accidents, consisting of sketches of instances of Extraction of Foreign Substances from the Natural Openings of the Human Body.* By R. Thompson, M. D., of Nashville, Tenn. (Read at a meeting of the Tennessee Medical Society, and ordered published by that Body.)

As no case has occurred in my practice, since we last met, of sufficient interest to be made the subject of a report, I have concluded as a substitute, to offer a condensed statement of a few cases of the above description which have occurred in the course of my past experience; not presented, however, because particularly novel, either in kind or management, but to fulfill my engagement to "report a case or read an essay," and to afford perhaps, a little momentary interest.

The first case I will introduce occurred in 1826, the first year I stood alone as a practitioner. A black bug of a species well known to the inmates of log tenements, of about the size of the end of one's little finger, exceedingly frisky and fond of running into holes, being out on an exploring expedition one night, and having a desire to examine the inner anatomy of the human cranium, or in search of something "good to eat," slipped himself into a boy's ear while asleep; but, like many other adventurers, he soon found a limit to his forward movement, and by reason of the tightness of the fit, was equally unable to "advance backwards." His ineffectual struggle for freedom soon awoke the boy to a consciousness of intense agony, extorting cries which brought an elder brother to his assistance, who having ascertained the difficulty, concluded to persuade the intruder to hold still by giving him a dram, and accordingly poured the meatus full of fourth proof, which soon had the desired effect, for the insect lay as quiet as a "bug in a rug," and permitted the boy to sleep till morning, when he was brought to me to have the thing extracted. But this was no easy matter—the forceps could not be made to grasp it without causing an amount of suffering which the little fellow would not bear. I accordingly devised an easier way: drawing the temper out of an iron knittingneedle, and having bent the end, hammered it into a very acute angle, and then by filing converted it into a spear with a barb. This I cautiously insinuated into the body of the insect, and managed to fix the barb into the hard substance which unites the wing to the body, which afforded a point sufficiently stable to enable me by gentle traction to extract the thing with but little suffering to the patient.

The next case I will describe occurred about two years subsequent to the above. A child of about a year was playing with its mother's thimble, and as children instinctively put everything in their mouths, this of course went there, and getting pretty far back and tickling the glottis, a spasmodic effort to vomit threw it above the soft palate immediately behind the septum nares. The family physician was sent for, who, seeing that the child breathed through both nostrils, decided that the thimble had been swallowed; but the mother not being satisfied, two other physicians from a neighboring county town were called in, who, after exploring the nares with a probe and meeting no thimble, also pronounced it swallowed, and put the child upon acids to dissolve it. But as its sufferings continued to increase I was sent for. From certain external signs—such as suffusion of the eyes—some enlargement about the root of the nose, &c., I became satisfied that the thimble must be in the nose, and as the other physicians had failed to detect it, I judged it must be in the position I have indicated. I gave my probe a curve to suit the duct of the nares, and then a side curve to bring the point behind the septum, and asked the mother if the doctors had used a probe of that form? She said they had not. I did this to satisfy her, she being opposed to my probing, as she said it would give the child unnecessary pain, "as the thimble was not there." With this instrument I found the thimble the first effort, and giving the probe to the mother, she too became certain from the firm resistance that the body was there, and became urgent that I should immediately extract it. But I knew that it was easier found than removed, and refused to make any effort until the family physician should be with me; who, by appointment met me the next day. I had prepared a strong iron probe with the necessary curves, square at the end, and ridged so as not to slip when brought to bear upon the edge of the thimble, the only chance being to push it into the fauces and extract it from thence. He undertook the task of dislodging it, while I stood ready with forceps in hand prepared to receive it the moment it should drop below the palate. After much difficulty he succeeded, but the moment it dropped below the palate the pharyngeal muscles seized it with such a spasmodic grasp that the hold of the forceps gave way twice without bringing it—in the mean time the child was dying of asphyxia, the glottis being entirely closed. In this extremity I threw away the forceps, and thrusting my finger forcibly into the thimble, and contracting the muscles so as to tighten it, and giving it a twisting tractile motion, was fortunate enough to dislodge it, much to my own gratification as well as that of the friends of the sufferer.

The next case I think worthy of notice occurred in 1835. A little fellow out of sheer wantonness pushed a large grain of corn up his nose. It was some hours before his father was made acquainted with the fact, and several more before I saw him; long enough to enable the grain to swell to near double its natural size, and imbed itself into the soft parts so that the forceps could not be made to grasp it. Again applying to the knitting-needle, I bent the end into a neat little hook, and pressing this sideways between the kernel and the septum, and then turning it, fastened the hook over the upper end of the grain, and now applying a thin delicately polished slip of reed to the other side, so as to enable it to slip, by a gentle drawing motion succeeded in dislodging it.

But shortly after this I met with another case which offered more difficulty. A number of children were amusing themselves hunting shells and round pebbles on a little island in a creek, and having found a very pretty oval pebble, conceived the idea of picking it up with the ear. One little girl in order to facilitate the operation, placed the pebble on its end and forced the other into the meatus; and while making some awkward efforts to extract it, pushed it further in until it rested upon the tympanum; every further effort was now the cause of suffering, and it was suffered to remain. The children being fearful of rebuke, failed to communicate the accident to the older members of the family until the parts had become much swollen and excessively sensitive. In this condition the body could not be grasped by the forceps without causing great pain, so I again resorted to invention. The spear would not do, as it could not penetrate the body, the hook would not answer, as the body was a smooth cone and offered no point upon which it could be fastened, so I concluded to snare it: so, procuring a wire which had helped to form the spring of an old fashioned suspender, I pushed a fold of it beyond the pebble, and holding one part in position by a slip of polished reed, by means of another brought the wire round the upper portion of the cone, and then by twisting the ends together made it embrace the pebble, so as to enable me to extract it with very little difficulty.

Since then I extracted a large bean from a little fellow's nose by the same snaring process.

The next case I will mention did not require much skill of execution, but a good deal of presence of mind and promptitude of action. While casually in a neighboring village to where I then lived, while walking down the street, a lady and two grown daughters came rushing out of their house, screaming most frantically, and exhibiting every other sign of intense mental agony and affright. I knew them well and demanded the cause of alarm, but failing to attract attention, I at once entered the apartment I had seen them emerge from, and passing on through open doors at length reached the nursery, where I found the youngest, a fine boy of some six months, laying on the floor apparently quite dead. Perceiving from the color that it was asphyxiated, I suspected it might be choked, and thrusting a finger into its mouth discovered a tough mass completely filling up the posterior fauces, and hermetically sealing the glottis. I soon succeeded in loosening and detaching it, and then set up artificial respiration by connecting its lungs with mine. After a few inflations I had the satisfaction of perceiving spasmodic efforts at respiration, and by the time its mother had screamed up a respectable crowd to condole with her for the loss of her youngest born, I was prepared to return it to her alive, and change the proffered sympathy into a burst of gratulation. In this case the child had been fed with clammy biscuit, which by a sucking motion of the tongue had been plastered upon the roof of the mouth, until by additions it excited a violent effort to swallow, but it was no go—a mere projection from the mass entered the esophagus, leaving the main body nicely fitting over the orifice of the wind-pipe.—*Jour. Med. and Surg.*

